# ASO Author Reflections: Essential to Reduce Adverse Outcomes in Perihilar Cholangiocarcinoma Surgery—Portal Vein Embolization

**DOI:** 10.1245/s10434-020-08333-9

**Published:** 2020-03-09

**Authors:** Pim B. Olthof, Thomas M. van Gulik

**Affiliations:** 1 Department of Surgery, Amsterdam UMC (Location AMC), University of Amsterdam, Amsterdam, The Netherlands; 2 Department of Surgery, Erasmus Medical Center, Rotterdam, The Netherlands

## Past

Major liver resection for perihilar cholangiocarcinoma is associated with 90-day mortality rates of up to 18% in Western series.^1^ Posthepatectomy liver failure is the primary cause of the high perioperative mortality.^2^ However, the golden standard preoperative procedure to reduce liver failure and mortality—portal vein embolization—is sparsely used in Western centers for patients with perihilar cholangiocarcinoma.^3^,^4^ This is in contrast to Eastern centers, where application of portal vein embolization is liberal and mortality rates are lower.^3^

## Present

Our recent paper shows that application of preoperative portal vein embolization before major liver resection for perihilar cholangiocarcinoma is associated with marked reductions in posthepatectomy liver failure and 90-day mortality rates. Using data of 1667 patients from 20 centers, a propensity score matched comparison was performed. In the matched cohort, liver failure and mortality rates were 8% and 7%, respectively, with resection after portal vein embolization, compared with 36% and 18%, respectively, after resection without portal vein embolization (*P* < 0.001 and *P* = 0.031).^4^ Although these results might be expected, these analyses have not been performed previously in a cohort with resections for only perihilar cholangiocarcinoma. The disease most often requires major liver resection as well as extrahepatic bile duct resection, which alone is high-risk surgery. But these patients often suffer from obstructive jaundice requiring biliary drainage, which can induce cholangitis and further increase the risk of adverse outcomes.

## Future

Perihilar cholangiocarcinoma is rare, and solid research on the optimal treatment strategy is difficult. In our opinion, a randomized trial on the effect of portal vein embolization is not required and perhaps unethical in light of the currently available evidence. Future studies should focus on the optimal indications for portal vein embolization. The goal is to provide a curative resection to as many patients as possible, while reducing the risks of adverse outcomes to a minimum. The balance between these two is a challenge for future studies. Perhaps alternative techniques such as quantitate liver function assessment can help to select those patients who will especially benefit from portal vein embolization.^5^
